# Robotic surgery for rectal cancer resection with complete intracorporeal double-stapling technique

**DOI:** 10.1007/s10151-024-02935-1

**Published:** 2024-05-18

**Authors:** J. Mazaki, T. Ishizaki, Y. Kuboyama, R. Udo, T. Tago, K. Kasahara, Y. Nagakawa

**Affiliations:** Department of Gastrointestinal and Pediatric Surgery, Tokyo Medical University, 6-7-1 Nishishinjuku, Shinjuku-ku, Tokyo, 160-0023 Japan

**Keywords:** Intracorporeal DST, Robotic surgery, Rectal cancer, Remote surgery

Recently, the adoption of robot-assisted surgery for rectal cancer has been increasing. External manipulation through a small umbilicus or Pfannenstiel incision is the mainstream to perform the anastomosis with EEA stapler. Herein, we report a robotic surgery for rectal cancer resection with complete intracorporeal double-stapling technique (CI-DST). Whether intracorporeal or extracorporeal anastomosis is planned, mechanical bowel preparation with isotonic polyethylene glycol and sennoside, and chemical bowel preparation with kanamycin and metronidazole are performed on the day before surgery. We will present the CI-DST technique in the low anterior resection (total mesorectal excision). Operative time was 272 min in this case.

There are some advantages in performing CI-DST. First, as the docking/undocking can be omitted, the ability to continuously maintain pneumoperitoneum allows for continuous surgical field development without the small intestine falling into the pelvis. Our standard port placement for rectal cancer resection is to place the camera port at the umbilicus using E-Z Access™ and Lap Protector™ (Hakko, Nagano, Japan) and remove the specimen from the same site. If a transverse low-abdomen incision (like Pfannenstiel) is used, it may be beneficial in terms of hernia reduction. Second, the stability and multi-articulation of robotic surgery enables us to perform intracorporeal dissection and suturing, which is easier compared to laparoscopic surgery. Third, before the anvil head is placed, blood flow can be confirmed by indocyanine green on both the proximal and distal sides simultaneously. Fourth, we believe this surgical procedure is advantageous for future remote surgeries, as there is no need for manipulation outside the body, allowing one remote surgeon and one local assistant to perform the anastomosis.

The disadvantage is the incision length, which is slightly longer than that with the natural orifice specimen extraction (NOSE) technique [1–3]. However, we believe that this technique is universal and is not affected by the site of the tumor or oncologic factors. It has been reported that NOSE can result in a slightly increased incidence of local recurrence for deep tumors (T4) [1, 4]. In addition, it is difficult to extract bulky tumors from the rectum. Furthermore, whether using the anastomosis with a circular stapler or single stapling technique, anastomosis cannot be performed after specimen removal unless a slightly longer distal margin is taken. Although there is no data with strong evidence for oncologic outcomes with robotic rectal surgery, this procedure may be an optimal alternative to extracorporeal anastomosis or NOSE, emphasizing the continuity with the conventional anastomosis that had been performed universally and can be used for any rectal cancer.

## Supplementary Information

Below is the link to the electronic supplementary material.

Supplementary file1 (MP4 236874 KB)

## Data Availability

This manuscript does not report data generation or analysis.
